# The Influence of Chemically Modified Potato Maltodextrins on Stability and Rheological Properties of Model Oil-in-Water Emulsions

**DOI:** 10.3390/polym10010067

**Published:** 2018-01-13

**Authors:** Karolina Pycia, Artur Gryszkin, Wiktor Berski, Lesław Juszczak

**Affiliations:** 1 Department of Food Technology and Human Nutrition, University of Rzeszow, Zelwerowicza 4 Str., 35-601 Rzeszow, Poland; kpycia@ur.edu.pl; 2 Department of Food Storage and Technology, Wroclaw University of Environmental and Life Sciences, Chełmońskiego 37 Str., 51-630 Wroclaw, Poland; artur.gryszkin@upwr.edu.pl; 3 Department of Carbohydrates Technology, University of Agriculture in Krakow, Balicka 122 Str., 30-149 Krakow, Poland; rrberski@cyf-kr.edu.pl; 4 Department of Food Analysis and Evaluation of Food Quality, University of Agriculture in Krakow, Balicka 122 Str., 30-149 Krakow, Poland

**Keywords:** maltodextrin, emulsion, stability, zeta potential, particle size, viscoelastic properties

## Abstract

The aim of this study was to determine the effect of the maltodextrins prepared from chemically modified starches (crosslinked, stabilized, crosslinked and stabilized) on the stability and rheological properties of model oil-in-water (o/w) emulsions. Based on the obtained results, it was concluded that emulsion stability depended on hydrolysates dextrose equivalent (DE) value. Maltodextrin with the lowest degree of depolymerization effectively stabilized the dispersed system, and the effectiveness of this action depended on the maltodextrin type and concentration. Addition of distarch phosphate-based maltodextrin stabilized emulsion at the lowest applied concentration, and the least effective was maltodextrin prepared from acetylated starch. Emulsions stabilized by maltodextrins (DE 6) prepared from distarch phosphate and acetylated distarch adipate showed the predominance of the elastic properties over the viscous ones. Only emulsion stabilized by maltodextrin prepared from distarch phosphate (E1412) revealed the properties of strong gel. Additionally, the decrease in emulsions *G*′ and *G*″ moduli values, combined with an increase in the value of DE maltodextrins, was observed.

## 1. Introduction

Oil-in-water (o/w) emulsions are the dispersed systems composed of an oil phase dispersed in an aqueous phase [1,2]. They create the basis for a wide range of natural and processed products, such as food, cosmetics, pharmaceuticals, biological fluids or fuels [3]. Dispersed phase molecules could be stabilized by the proteins, polysaccharides or emulsifiers [4,5]. Such types of dispersions have a tendency to be thermodynamically unstable. Emulsion instability is connected by two physical processes. The first one is to increase the dimensions of the dispersed phase particles due to flocculation or coalescence; the other is the migration of particles leading to creaming or sedimentation [2,6,7]. The visual effect of these processes is a clear phase separation of the emulsion. Therefore, the particle size of the dispersed phase, and the occurrence frequency of given particle sizes could be considered as the index of emulsion stability [6]. The leading method of minimizing the rate of emulsion destabilization is the proper selection of emulsifier or/and stabilizing agents, the proper level of system dispersion to minimize the differences in density of the phases by the addition of an aggravating agent to the oil phase, or to increase the viscosity of the continuous phase and, as a consequence, cause the limitation of the molecular mobility of dispersed phase [7]. Emulsifiers stabilize emulsion for a short period of time; a much longer emulsion stability is related to the addition of stabilizing substances, such as polysaccharides [8]. The action of polysaccharides relies on an increase of the continuous phase viscosity, causing the reduction of molecular mobility of the dispersed phase [2]. The increase in the hydrocolloids concentration intensifies the emulsion stabilizing effect. According to some authors [9,10,11] the stabilizing effect of acacia, xanthan or guar gums can be obtained even at concentrations lower than 0.1%. The literature also provides examples of the application of maltodextrins, derived from native starches of different botanical origin, as stabilizers of o/w food emulsions [8,12,13,14]. Maltodextrins are manufactured by enzymatic starch hydrolysis by amylolytic enzymes. The universal indicator of their properties is a dextrose equivalent (DE) [15]. Starch hydrolysis products, concentrating the continuous phase of o/w emulsion, not only affect the stability of such systems but also their rheological properties [12]. So far, in studies focused on emulsion stability, except for hydrocolloids used as stabilizers, a synthetic emulsifier Tween 80 was also applied [2,12]. The stabilizing effect relies on the synergistic action of the emulsifier and polysaccharide hydrocolloid thickening the continuous phase.

The literature provides limited information about improved utility and rheological properties of o/w emulsions due to addition of maltodextrins prepared from chemically modified starches. As shown by Pycia et al., [16] native potato starch-based maltodextrin was able to stabilize foams. However, its effectiveness depended on the hydrolyzate DE value, and its share in the system. Moreover, 30% addition of potato starch-based maltodextrin with DE 6 guaranteed the stability of albumin foam. In the case of maltodextrins derived from chemically modified starches, the nature of chemical starch modification additionally determined the hydrolysates properties. Hence, it should be assumed that such maltodextrins show different action in dispersion systems, depending on the type of starch chemical modification, which was applied to create their matrix. 

Therefore, the aim of this study was to determine the effect of maltodextrins, prepared from chemically modified starches (crosslinked, stabilized, and both crosslinked and stabilized), different applied concentrations and DE values on the stability and the rheological properties of model o/w emulsions.

## 2. Materials and Methods 

### 2.1. Materials

The maltodextrins (M) used as stabilizers were produced from the following chemically modified starches: distarch phosphate (E1412), acetylated distarch phosphate (E1414), acetylated starch (E1420) and acetylated distarch adipate (E1422) (WPPZ S.A., Luboń, Poland). The maltodextrins with DE 6, 11 and 16 were obtained by laboratory enzymatic method using a bacterial α-amylase (BAN 480 L, Novozymes, Bagsvaerd, Denmark), and hydrolysis times were established experimentally [17]. Dextrose equivalent (DE) of the obtained maltodextrins was determined by Schoorl-Regenbogen method [17].

The research material was the model o/w emulsion. Continuous phase was the aqueous solution of the respective maltodextrin, prepared from chemically modified starch, at a given concentration and degree of saccharification, and sunflower oil (Kruszwica, Poland) created a dispersed phase. The ratio of the dispersed phase to the continuous one in the analyzed emulsions was 1:4. Solutions of maltodextrins with concentrations between 10–50% *w*/*w* (depending on the type of maltodextrin) for DE 6 maltodextrins, whereas for DE 11 and 16 within 10%–60% range were prepared at 25 °C. Next, the appropriate amount of vegetable oil was added to the prepared maltodextrin solution and the whole was homogenized for 2 min at 9500 rpm using Ultra-Turrax T 25 homogenizer (IKA-Werke, Staufen, Germany).

### 2.2. Methods

#### 2.2.1. Evaluation of Emulsion Stability

Determining the maltodextrin ability to stabilize the model o/w emulsion was performed by placing the emulsion samples into a glass tube, and observing the amount of separated free oil phase. Observations were made at 25 °C for 7 days. The ability to stabilize the emulsions was expressed as a creaming index (CI) defining a degree of the oil phase binding in time:

(1) $$\mathrm{CI}=\frac{H_{L}}{H_{E}}\;\cdot \;100\%$$

where: CI—creaming index (%), *H*_L_—the height of the serum layer formed at the bottom of glass tubes, *H*_E_—the height of total emulsion layer (mm).

#### 2.2.2. Zeta Potential of o/w Emulsion 

The ζ potential of emulsion droplets was determined by the microelectrophoretic method, using Malvern Zetasizer Nano ZS apparatus (Malvern Instruments, Worcestershire, UK). Each value was calculated as an average from three consecutive measurements with 20 runs. The ζ potential measurements were performed at 25 °C. 

#### 2.2.3. Particle Size Distribution (PSD) of Oil Droplets in Maltodextrins Stabilized o/w Emulsions

PSD of oil droplets in model o/w emulsions stabilized by maltodextrins solutions were performed by means of laser diffraction method using Mastersizer 2000 apparatus (Malvern Instruments) [18]. 0.5 g of freshly prepared emulsion was dissolved in a beaker in 150 cm^3^ of 0.1% sodium dodecyl sulfate (SDS) solution and stirred using a magnetic stirrer for 15 min at 500 rpm. SDS solution was added to prevent rapid destabilization of the viscosity-stabilized dispersion after dilution. In the first stage of the analysis the background obscuration (distilled water) was measured. Next, measurement of oil droplets of particle size in the model emulsion systems was performed. The final result was presented in the form of a volume average diameter *d*_4,3_, and as the figure percentage of defined size particles fraction (µm). For emulsions stabilized by DE 6 maltodextrins the measurements of oil droplets size was also performed after one week of storage.

#### 2.2.4. Rheological Properties Analysis of Model o/w Emulsions

Mechanical spectra of model emulsions prepared from 50% maltodextrin solutions were performed at 25 ± 0.5 °C using cone–plate configuration (cone diameter 35 mm, angle 1, gap size 0.105 mm). The measurements were done at linear viscoelasticity range, at constant strain amplitude equal to 0.01 and angular frequency within 1 to 100 rad/s range. Resulting curves were described by power equations:

*G*′ = *K*′ · *ω^n^*^′^ (2)

*G*″ = *K*″ · *ω^n^*^″^ (3)

where: *G*′—storage modulus (Pa), *G*″—loss modulus (Pa), *ω*—angular frequency (rad/s), *K*′, *K*″, *n*′, *n*′—constants.

Measurements were performed in triplicate.

### 2.3. Statistical Analysis

In order to establish the statistical differences between means the data were treated by one-factor or two-factor ANOVA, and the least significant difference (LSD) using Duncan test at significance level 0.05 was calculated. Calculations were performed using statistical software package Statistica v.12 (StatSoft Inc., Tulsa, OK, USA).

## 3. Results

### 3.1. Emulsions Stability

O/W emulsion systems tended to be thermodynamically unstable due to the occurrence of such processes as flocculation, coalescence or phase separation (creaming). In order to counteract such negative phenomena, the hydrocolloids were applied managing the stability problems of the emulsion, mainly by affecting the rheological properties of these types of systems [2]. Maltodextrins used as a stabilizer thickened the emulsion continuous phase, and also at the same time modified the rheological properties of such systems [12]. However, as shown by this study, the character of changes depended on the maltodextrin type, its degree of saccharification and concentrations in the system [1,12]. In the case of DE 6 maltodextrins-based emulsion, the largest variation was observed due to the concentration and type of hydrolyzate stabilizing the model dispersion system. 

**Figure 1:**
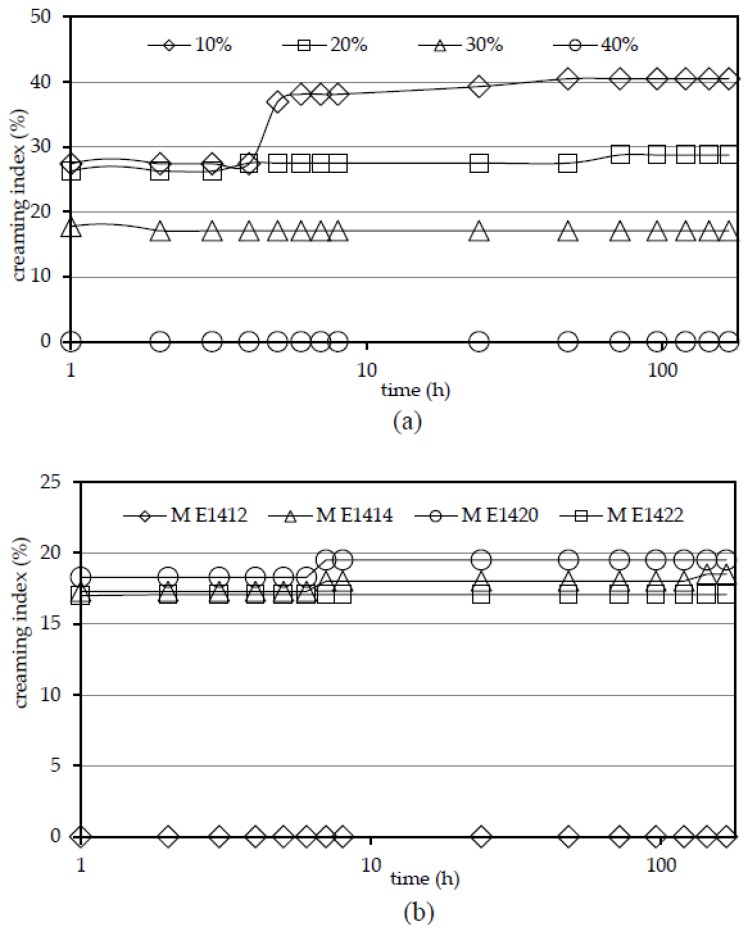
Creaming index of o/w emulsions with different concentration of M E1422 (DE 6) addition (**a**); and with addition different maltodextrins (DE 6) at 30% concentration (**b**).

Figure 1a,b show the stability of the emulsion, expressed as CI values influenced by addition of M E1422 at different concentrations (Figure 1a), and under addition of different maltodextrins of (DE = 6) at 30% concentration (Figure 1b). Creaming index values decreased with increasing concentration of maltodextrin (Figure 1a), but the maltodextrin concentration at which no phase separation was observed depended on its type. It was observed that maltodextrin prepared from distarch phosphate (M E1412) stabilized emulsion at as low concentration as 30%. The lowest ability to stabilize emulsion was observed for acetylated starch-based maltodextrin (M E1420), because only the emulsion with the addition of the maltodextrin solution of DE 6 and 50% was stable. Such emulsion behavior could result from a low viscosity of analyzed maltodextrin solution [19]. It is likely that the need to use such high maltodextrin concentrations in order to stabilize the emulsion was due to the chemical nature of the applied hydrolyzate. Additional functional groups present in M E1420, introduced by starch acetylation process, reduced glucose chains aggregation creating a weaker structure of continuous phase and resulting in a greater mobility and susceptibility of oil droplets to aggregate. For the remaining maltodextrins prepared from crosslinked and stabilized starches (M E1414 and M E1422) their 40% solutions gave stable emulsions for a one-week storage period. For DE 6 maltodextrins the ability to stabilize the emulsion was different, and depended upon the chemical nature of maltodextrin. On this basis, the maltodextrin prepared from chemically modified starches can be ordered by their ability to stabilize o/w emulsions: M E1412 > M E1414 = M E1422 > M E1420. In the case of hydrolysate prepared from crosslinked starch, the creation of additional bonds in the polymer structure resulted in greater ability to thicken the continuous phase. Thus, it appears, that such high concentration of M E1420, at which the system was stable, resulted from the fact that the additional acetyl groups created during starch modification impeded the chains association and the formation of a compact structure capable of retaining oil droplets. In the case of double-modified starch-based maltodextrins (M E1414 and M E1422) there was no difference between them in terms of ability to prevent emulsion separation.

An attempt was also undertaken to stabilize the model emulsion by means of maltodextrin with a higher degree of saccharification, i.e., DE values of 11 and 16. However, despite the addition of these maltodextrins, prepared from different modified starches, in a relatively short time after preparation the phase separation occurred with a visible upper oil phase. The destabilization was observed even in the system containing 60% maltodextrin solution. This type of behavior was due to the low viscosity of DE 11 and 16 maltodextrins solutions, caused by advanced hydrolysis process [19]. Therefore, in the case of hydrolysates with DE 11 and 16 no effect of the type of chemical starch modification, of being a matrix for the maltodextrins creation, and for their ability to stabilize the dispersions was observed. According to Dokic-Baucal et al. [12] the emulsion stability with the addition of a maltodextrin solution, acting as a stabilizing agent, depended on the starch hydrolysis extent, because low DE values hydrolysates had a higher content of high molecular fractions resulting in formation of a three-dimensional structure, which prevented the release of oil from the emulsion. A similar relationship was observed by Udomrati et al. [2], who studied the stability of the emulsion based on solutions of tapioca maltodextrins. However, according to Lewandowicz et al. [14] maltodextrins regardless of their properties (preparation method, the degree of saccharification and dispersion concentration in the system) had no emulsifying properties, and their emulsions separated immediately at the end of homogenization. In the studied cases it was found that only DE 6 hydrolysates had the ability to stabilize the emulsion, but as hypothesized, the effect was mostly due to viscosity increase of the continuous phase, and not due to the properties of emulsifiers.

### 3.2. Emulsions ζ Potential

In the investigated model emulsion systems containing DE 6 maltodextrins, which were stable for a week storage period, the ζ potential was analyzed. The ζ potential is an important parameter allowing description of the behavior of hydrocolloids in dispersion systems [20]. This parameter is applied for the numerical description of the dispersion system stability, and is determined on the basis of molecular mobility in the electric field. It is assumed that for the stable emulsion the absolute value of the ζ potential is greater than 30 mV (ζ > +30 mV or ζ < −30 mV). The greatest influence on the ζ potential value has emulsion pH value. The alteration of pH could change the potential value from the positive to negative [6]. The values of the ζ potential of a stable emulsion with addition of various DE 6 maltodextrins are summarized in Table 1. 

The value of ζ potential of the emulsion immediately after preparation (0 day), depended on the type of hydrolyzate, ranged from 15.1 mV (M E1414) to 18.3 mV (M E1422). With increasing storage period the values of this parameter did not significantly change, as at the last day of observation (7 day) it varied from 14.5 mV (M E1414) to 19.1 mV (M E1422). Independently of the storage time, the smallest values of the ζ potential were observed for the emulsion with M E1414 addition, and the highest for dispersion systems containing M E1420 and M E1422. According to Mirhosseini et al., [21] ζ potential of the emulsion strongly depends on the type and concentration of the polysaccharide applied to stabilize system. Cited authors observed an increase in ζ potential values of fruit beverages with increasing concentrations of xanthan gum used as stabilizer. In their opinion, an increase in the concentration of stabilizing agent led to the accumulation of negative charges, resulting in a negative value of system ζ potential. Nevertheless, Klinkesorn et al. [13] observed decline of ζ potential values of the emulsion with increasing maltodextrin concentration in the system. The mentioned authors turned their attention to the high ζ potential values (~−40 mV) emulsion despite the presence of a non-ionic stabilizer, which was maltodextrin. Similar results were also obtained by other researchers [22]. According to Hsu and Nacu [22] the value of the ζ potential of the emulsion droplets, stabilized by non-ionic formulations, was due to the adsorption of H_3_O^+^ (low pH) or OH^−^ ions (high pH) from the water. The value of emulsion ζ potential could be also influenced by the negative charge resulting from the presence of free fatty acids and phospholipids in the oil. Moreover, Klinkesorn et al., [13] observed a slight decrease in the ζ potential value with increasing maltodextrin concentration, and a decrease of DE value, probably as a result of the increased hydrolysate concentration, followed by the binding of maltodextrins with surfactans that, in the absence of hydrolyzate, were associated with oil droplets. Therefore, the reduction of ζ potential was observed. However, this phenomenon limited the DE of the hydrolyzate since, as shown by Waganslant et al. [23], maltodextrin–surfactant bonds occurred when the hydrolysate chain length was more than 12 units. Furthermore, Abdolmaleki et al. [20] found, that ζ potential value of the o/w emulsion containing tragacanth gum and sodium chloride decreased with increasing concentration of sodium chloride, and with a decrease of system pH. According to cited researchers this phenomenon relied on a decrease in the electrostatic interactions caused by increasing NaCl concentration.

### 3.3. Analysis of Oil Droplets Size

From a thermodynamic point of view, a reduction of the stability of o/w type dispersion is related to the increasing size of the dispersed phase particles. Hence the size of the particles of the dispersed phase, and frequency of occurrence of a certain size particles fractions can be regarded as an indicator of emulsion stability [6]. The size of the dispersed phase particles is related to the system viscosity and composition (the presence of an emulsifier, the proportions of phases) the main factor determining the o/w emulsion stability [6,18]. The dispersed phase particle size depends on the rate and duration of homogenization, the presence of emulsifiers, pH and system viscosity [23]. In the aqueous dispersion phase, with a relatively low viscosity, the oil phase is easily dispersed. 

Table 2 summarized the average diameter of oil droplets of the stable emulsion prepared with addition of DE 6 maltodextrins. The only droplet size increase with the concentration rise was observed in M E1412 and M E1422, from 10% to 20% maltodextrin concentration; for all the other concentrations the droplet size reduces with the increase concentration (Table 2). The increase in the concentration of maltodextrin in the system increases the viscosity of the continuous phase, which makes it difficult to combine the droplets into larger units, which prevents destabilization of the emulsion by flocculation or coalescence. In emulsions prepared with the use of 10% maltodextrin solutions, a statistical variation in terms of average particle diameters of the dispersed phase was observed. The smallest oil droplets were found in M E1412-based emulsion, and the largest in the emulsion were found with the addition of M E1420. As previously demonstrated, the maltodextrin concentration providing the emulsion with proper stability depended on their nature. Among all stable emulsions the smallest-diameter values of the dispersed phase were observed for M E1412. The highest average particle diameters of the dispersed phase were found in the emulsion prepared with addition of 10% solution of acetylated starch-based maltodextrin (M E1420), but the discussed dispersion proved to be stable only under the influence of 50% maltodextrin solution. Such high hydrocolloid concentrations could cause the reduction of the particle size in the dispersed phase (Table 2).

Analysis of the distribution size of oil droplets in emulsions containing DE 11 and 16 maltodextrins revealed a decrease in the size of the oil droplets with increasing maltodextrin concentrations. Table 3 summarized the average diameter of oil droplets in emulsions stabilized by DE 16 maltodextrins. However, despite the small size of the oil droplets, the analyzed dispersions with the addition of stabilizing agents in the form of DE 11 and 16 maltodextrins underwent a separation process in a relatively short period of time (about 60 min) after the end of homogenization.

**Figure 2:**
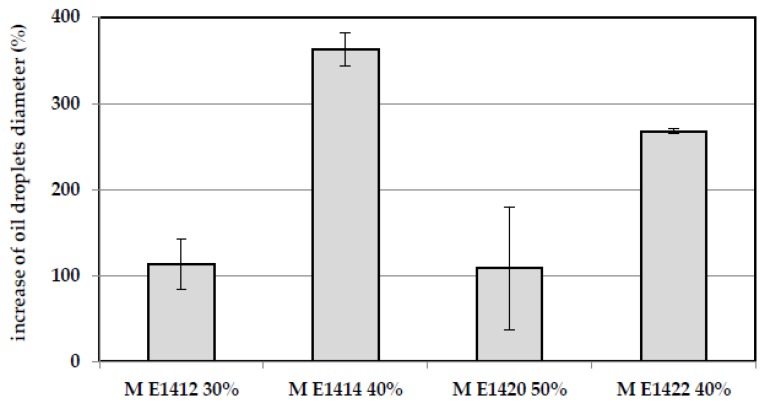
The increase of oil droplets diameter in DE 6 maltodextrins-based emulsions after a week storage.

The size of the oil droplets in maltodextrin-stabilized emulsions resulted, among other things, from the compact structure of the oil phase in the emulsion due to the high concentration of the continuous phase. According to Ye et al. [11] as the hydrocolloid concentration increased, the size of the oil droplets decreased, caused by high concentration of the continuous phase. Moreover, the smaller size of the oil droplets associated with high maltodextrin level results resulted from a higher availability of maltodextrins absorbing themselves on the surface of oil droplets, thereby preventing their aggregation. Additionally, increasing concentration of maltodextrins in the analyzed model emulsions led to an increase in viscosity of the emulsion continuous phase. Thus, high viscosity contributed to a reduced mobility of the oil droplets, which, in extreme cases, caused their binding into larger agglomerates, and, as an effect, resulted in an increase of their average diameter [24]. A similar trend in oil particle size distribution in emulsions was observed by Tzoumaki et al. [25] analyzing particle size distribution of chitin nanoparticle-stabilized emulsion. As indicated earlier, some emulsions stabilized with the maltodextrin solutions with the lowest degree of depolymerization showed no separation over a week storage period, which proved the stability of this type of system. However, despite the lack of visual separation of the oil phase, the thermodynamic nature of the stability associated with the size of oil droplets must be also examined. Despite the visual stability during a week-long storage period of the emulsion prepared from DE 6 maltodextrins, an increase in the size of oil micelles of the individual emulsion was noted. This probably indicated a lack of thermodynamic stability of such systems that were visually stable. However, the observed size of the oil droplets was not able to sufficiently separate the emulsion oil phase. The percentage increase of the size of the oil droplets after seven days of storage was shown in Figure 2. After a week of storage, the highest increase in diameter of the micelles was found in emulsions stabilized by 40% solutions of dual-modified starch-based maltodextrin (M E 1414). In the case of emulsions containing solutions of distarch phosphate-based maltodextrins (M E1412) an increase in the size of the oil droplets after a storage period was documented, but after six days of storage the size of the lipid droplets doubled.

### 3.4. Rheological Properties of Emulsions with Addition of Different Maltodextrins

Information on the rheological properties of emulsions is important from a food design point of view. Creaminess, smoothness, spreadability or fluidity are just some food product features that are closely related to the rheological properties. Viscosity is a basic rheological parameter determining the stability of the emulsion. Moreover, it affects other emulsion characteristics, such as the size and distribution of the dispersed phase, the extent of dispersion, or the occurrence of interaction among the phase components [26].

**Figure 3:**
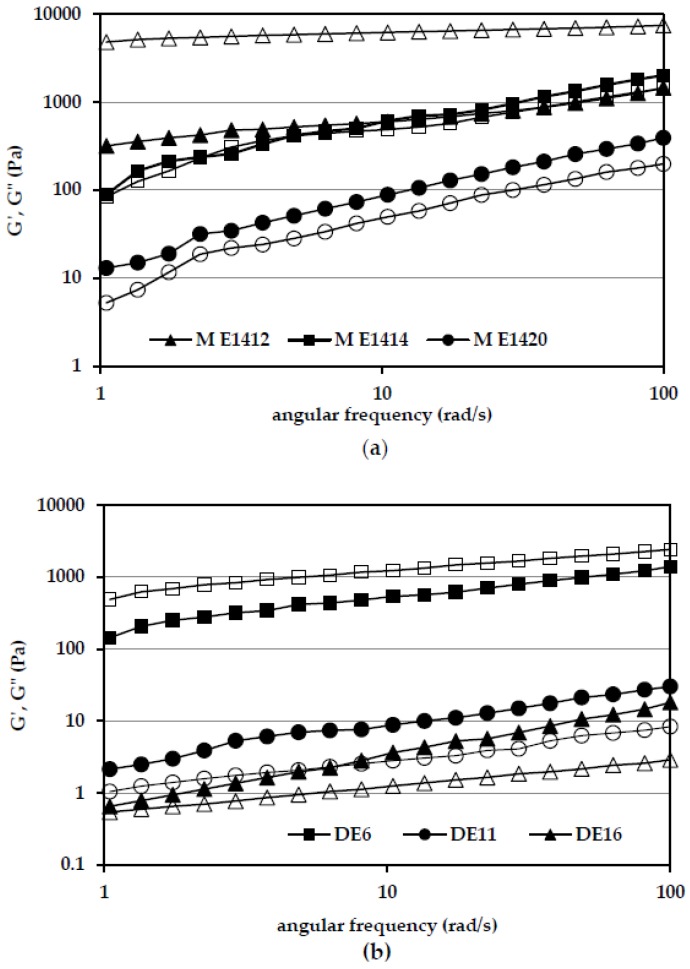
Mechanical spectra of emulsions with addition of 50% solutions of selected DE 6 maltodextrins (**a**), and with addition of 50% solution of DE 6–16 M E1422 (b). *G*′—empty markers, *G*″—filled markers.

Figure 3a,b present the mechanical spectra of the tested emulsion systems prepared from 50% solution of maltodextrins originating from a various starches, and with addition of DE 6–16 M E1422. The obtained results proved that dispersions stabilized by DE 6 maltodextrins prepared from E1412 and E1422 revealed a predominance of the elastic (*G*′) properties over the viscous (*G*″) ones. However, in emulsions containing the remaining maltodextrins, viscous properties predominated over elastic properties (Figure 3a). The clear superiority of *G*″ modulus over *G*′ one was found in the emulsion with the participation of M E1420. The greatest value of storage modulus was found for an emulsion with the addition of distarch phosphate-based maltodextrin (M E1412), and lowest for emulsion stabilized with the addition of acetylated starch-based maltodextrin (M E1420). This confirmed the results of studies on the stability of this type of systems. The most stable emulsion was characterized by the highest values of moduli *G*′ and *G*″. The rheological properties of the emulsion resulted from the character of chemical starch modification, which was maltodextrin matrix. Bortnowska et al. [27], analyzing the viscoelastic properties of the model emulsions stabilized with the addition of pregelatinized waxy corn starch, showed an increase in *G*′ and *G*″ moduli values with increasing concentrations of polysaccharide in the system. According to the cited authors [27], as the concentration of the hydrocolloid in system was growing, a three-dimensional structure was formed, with gel-like properties. 

Figure 3b shows, based on M E1422 containing emulsions, the influence of the degree of maltodextrin saccharification on the rheological properties of the emulsion. The greatest values of storage and loss modulus were found for an emulsion stabilized by maltodextrin with the lowest degree of depolymerization. The magnitude of emulsion *G*′ and *G*″ moduli decreased with an increase in the hydrolysates DE value. It resulted from the maltodextrin viscosity that, as confirmed by numerous studies [2,12,19], decreases as the dextrose equivalent (DE) increased.

**Figure 4:**
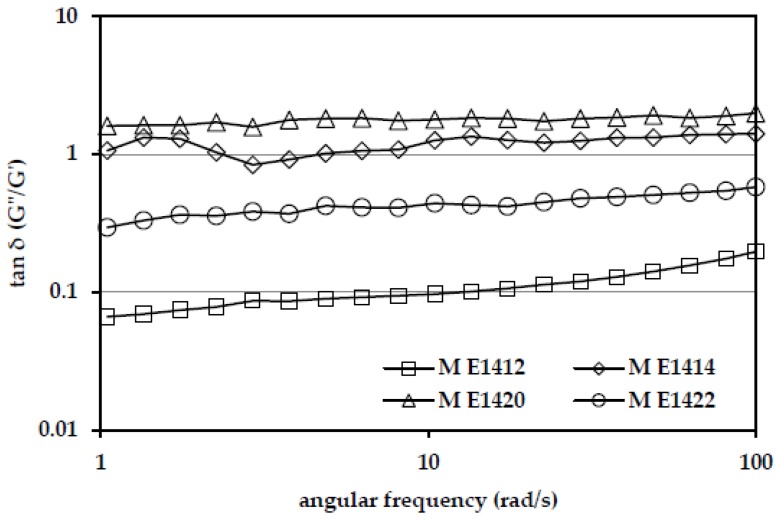
The values of the tangent of the phase angle (δ) depending on the angular frequency for the emulsion stabilized with selected DE 6 maltodextrins at different concentrations (M E1412—30%; M E1414, M E1422—40%; M E1420—50%).

Analysis of the magnitude and relative proportions of the storage and loss modulus allowed specification of the mechanical nature of the system. The ratio of the energy loss to stored in each cycle was described as the tangent of the phase angle (tan δ), which provided information about the physical behavior of the system [27]. Figure 4 shows the values of the phase angle tangent for the emulsion prepared from the selected DE 6 maltodextrins. The tangent of the phase angle (tan δ = *G*″/*G*′) values indicated that the emulsion stabilized by DE 6 distarch phosphate maltodextrin (M E 1412) to a certain value of the angular frequency (about 10 rad/s) had a nature of a strong gel. At higher values of the angular frequency its properties changed into that typical of a weak gel. At the same time, emulsions containing solutions of the other DE 6 maltodextrins behaved like weak gels. Emulsions containing DE 11 and 16 hydrolysates were typical for the dispersions properties (tan δ > 1), which results from a degree of hydrolysis and properties of maltodextrins used as stabilizers. The majority of short-chain fractions resulted in solutions with low viscosity, which determined the impossibility to form a typical strong gel structure. Emulsions with 5% addition of pregelatinized waxy corn starch were characterized by typical weak gel-like properties, such as for dressings and mayonnaise [27]. 

Table 4 provides the parameters of a power model used to describe the mechanical spectra of the investigated emulsions stabilized by 50% maltodextrin solutions. Two-factor analysis of variance proved that both the type of maltodextrin and its degree of saccharification had a significant impact on the viscoelastic properties represented by the parameters of the power type equation described the mechanical spectra. In the case of the dispersion systems with the addition of DE 6 maltodextrin solutions, the value of *K*′ parameter providing information about the initial value of the *G*′ modulus varied over a wide range from 16.01 (M E1420) to 5026.13 Pa (M E1412), and the value of *K*″ parameter indicating the magnitude of *G*′ modulus ranged from 25.98 (M E1420) to 319.94 Pa (M E1412). Only in the case of M E1420 containing emulsion was a higher value of the parameter *K*″ in relation to *K*′ observed, indicating the superiority of the viscous properties over elastic ones. The values of *n*′ and *n*″ parameters indicating the sensitivity of the moduli to the angular frequency change showed a statistical difference. The highest values of *n*′ and *n*″ parameters were calculated for M E1420 containing emulsion. In the case of dispersions, where the continuous phase constituted a solution of DE 11 maltodextrins, a clear decrease in the values of the parameters *K*′ and *K*″ was noted (Table 4). Similarly, as in case of DE 6 hydrolysates stabilized emulsions, M 1420 showed the lowest values of *K*′ and *K*″ parameters. In the case of emulsions in which the disperse phase was created by a solution of maltodextrin prepared from a dually modified starch a distinct superiority of viscous properties over elastic ones was discovered. The application of DE 16 maltodextrins as stabilizers resulted in a further drop in the power-law model parameters. A distinct predominance of the viscous properties over the elastic ones was found in the case of M E1420 emulsion. In addition, attention was drawn to high values of the *n*′ and *n*″ which indicated a high sensitivity of *G*′ and *G*″ moduli on angular frequency changes. The decrease in the value of the *K*′ and *K*″ parameters for emulsions with increasing values of dextrose equivalent resulted from apparent viscosity of the hydrolysates solution, which decreases with increasing DE, which was confirmed by several studies [2,19]. Pycia et al. [16], examining the effect of the concentration and saccharification degree of potato maltodextrin on rheology of albumin foams, also observed a decrease of *K*′ and *K*″ parameters values as the DE of the hydrolyzate increased. Lewandowicz et al., [14] attempted to apply a DE 9 and 25 potato maltodextrin as a mayonnaise-stabilizing agent. They confirmed the influence of the hydrolysates addition on the rheological properties of this type system. A predominance of elastic properties over viscous ones, and a decrease in the values of *G*′ and *G*″ moduli with increasing values of hydrolysates DE were observed. At the same time, the investigated mayonnaise showed the properties of a weak gel, regardless of the type of stabilizing maltodextrin. 

## 4. Conclusions

The application of maltodextrins prepared from chemically modified starches in model o/w emulsions influenced the stability and the rheological properties of these systems. The nature of the observed changes depended on the maltodextrin type (a type of chemical modification of the starch, which created a matrix to obtain them), the dextrose equivalent (DE) and concentration at which it occurred in the system. It was proven that only DE 6 maltodextrins stabilized the model o/w emulsions, and this effectiveness depended on maltodextrin type and their concentration in the system. It was documented that distarch phosphate-based maltodextrin was the most effective emulsion stabilizer, but the least acetylated starch-based maltodextrin. The values of the ζ potential of the emulsion during a week of monitoring did not significantly change, which confirmed the stability of the studied systems.

However, in emulsions stable for a week storage period the growth of the oil droplets diameter in dispersed phase was observed, the largest being in emulsions with the addition of maltodextrin prepared from dually modified starches. Despite this fact, no visual destabilization of the system was observed. Emulsions stabilized by DE 6 maltodextrins prepared from E1412 and E1422 showed a predominance of the elastic properties over viscous properties, the other ones the modulus dependence. *G*′ and *G*″ moduli values decreased with the increase of maltodextrin DE value. Only the emulsion with the addition of DE 6 M E1412 showed properties of a strong gel.

## Figures and Tables

**Table 1:** Changes of the ζ potential values of emulsions with addition of DE 6 maltodextrin during storage.

Type of Maltodextrin	ζ Potential (mV)		
	Day of Storage		
	0	3	7
M E1412	−16.9 ^b^ ± 1.1	−17.2 ^c^ ± 2.1	−16.4 ^b^ ± 1.5
M E1414	−15.1 ^a^ ± 1.2	−15.8 ^a^ ± 1.3	−14.5 ^a^ ± 0.8
M E1420	−17.0 ^b^ ± 2.4	−16.8 ^b^ ± 1.1	−17.6 ^c^ ± 2.1
M E1422	−18.3 ^c^ ± 1.1	−18.5 ^d^ ± 1.2	−19.1 ^d^ ± 1.3

Mean values ± standard deviation in column marked with the same superscript do not differ significantly at significance level of 0.05. The concentrations of maltodextrins M E1412, M E1414, M E1420 and M E1422 solutions were 30%, 40%, 50% and 40%, respectively.

**Table 2:** Average diameter of oil droplets in freshly prepared emulsions stabilized by DE 6 maltodextrins solutions.

Type of Maltodextrin	*d*_4,3_ (µm)				
	Concentration (%)				
	10	20	30	40	50
M E1412	181.4 ^a^ ± 0.8	234.2 ^b^ ± 8.7	101.3 ^a^ ± 0.7	-	-
M E1414	271.6 ^c^ ± 1.9	236.0 ^b^ ± 4.7	197.8 ^d^ ± 3.1	81.3 ^a^ ± 0.7	-
M E1420	275.0 ^c^ ± 3.8	213.6 ^a^ ± 2.4	190.1 ^d^ ± 1.8	102.5 ^c^ ± 1.2	66.4 ^a^ ± 1.9
M E1422	206.9 ^b^ ± 2.9	241.8 ^c^ ± 8.3	107.6 ^b^ ± 0.6	86.8 ^b^ ± 0.6	-

Mean values ± standard deviation in column marked with the same superscript do not differ significantly at significance level of 0.05.

**Table 3:** Average diameter of oil droplets in freshly prepared emulsions stabilized by DE 16 maltodextrins solutions.

Type of Maltodextrin	*d*_4.3_ (µm)					
	Concentration (%)					
	10	20	30	40	50	60
M E1412	191.1 ^c^ ± 8.0	143.1 ^b^ ± 3.7	128.3 ^c^ ± 5.1	115.7 ^c^ ± 4.8	54.7 ^b^ ± 0.1	46.0 ^c^ ± 0.6
M E1414	126.2 ^a^ ± 2.5	117.0 ^a^ ± 1.3	120.3 ^b^ ± 2.1	108.5 ^b^ ± 1.2	56.5 ^b^ ± 1.1	47.5 ^c^ ± 2.7
M E1420	121.5 ^a^ ± 4.8	116.6 ^a^ ± 0.8	90.3 ^a^ ± 0.9	137.2 ^d^ ± 7.1	71.7 ^c^ ± 3.4	46.3 ^c^ ± 0.8
M E1422	135.6 ^b^ ± 2.4	120.5 ^a^ ± 2.9	137.3 ^c^ ± 1.4	132.4 ^d^ ± 3.0	47.2 ^a^ ± 0.5	40.3 ^a^ ± 0.6

Mean values ± standard deviation in column marked with the same superscript do not differ significantly at significance level of 0.05.

**Table 4:** Parameters of power model applied to describe mechanical spectra obtained for emulsions stabilized by 50% maltodextrin solutions.

Type of Maltodextrin	*K*′	*n*′	*R*^2^	*K*″	*n*″	*R*^2^
	**DE 6**					
**M E1412**	5026.13 ^d^ ± 99.53	0.08 ^e^ ± 0.01	0.9938	319.94 ^e^ ± 11.60	0.28 ^f^ ± 0.03	0.9783
**M E1414**	134.93 ^b^ ± 2.51	0.53 ^c^ ± 0.01	0.9402	138.29 ^c^ ± 0.47	0.59 ^a, b, c^ ± 0.01	0.9751
**M E1420**	16.01 ^a^ ± 2.66	0.62 ^d^ ± 0.10	0.9932	25.98 ^b^ ± 1.92	0.63 ^b^ ± 0.07	0.9970
**M E1422**	603.14 ^c^ ± 19.50	0.30 ^a^ ± 0.02	0.9882	197.37 ^d^ ± 10.33	0.42 ^d^ ± 0.04	0.9889
	**DE 11**					
**M E1412**	3.78 ^e^ ± 0.12	0.38 ^a, b^ ± 0.01	0.9794	3.75 ^a^ ± 0.24	0.47 ^d^ ± 0.01	0.9925
**M E1414**	2.63 ^d^ ± 0.28	0.53 ^c^ ± 0.03	0.9759	5.82 ^a^ ± 0.48	0.18 ^e^ ± 0.02	0.9811
**M E1420**	2.28 ^c^ ± 0.09	0.34 ^a^ ± 0.01	0.9293	2.06 ^a^ ± 0.04	0.61 ^a, b^ ± 0.01	0.9735
**M E1422**	1.04 ^b^ ± 0.01	0.43 ^b^ ± 0.01	0.9889	2.44 ^a^ ± 0.09	0.54 ^c^ ± 0.01	0.9819
	**DE 16**					
**M E1412**	1.76 ^c^ ± 0.56	0.44 ^b^ ± 0.01	0.9869	1.51 ^a^ ± 0.05	0.57 ^a, c^ ± 0.01	0.9947
**M E1414**	0.54 ^a, b^ ± 0.04	0.60 ^c, d^ ± 0.02	0.9723	1.36 ^a^ ± 0.15	0.98 ^h^ ± 0.02	0.9875
**M E1420**	0.73 ^b^ ± 0.10	0.62 ^d^ ± 0.01	0.9921	0.77 ^a^ ± 0.08	0.62 ^a, b^ ± 0.02	0.9872
**M E1422**	0.52 ^a, b^ ± 0.02	0.36 ^a, b^ ± 0.00	0.9983	0.63 ^a^ ± 0.01	0.71 ^g^ ± 0.02	0.9980
**Two-Factor ANOVA—*p***						
**factor 1**	<0.001	<0.001		<0.001	<0.001	
**factor 2**	<0.001	<0.001		<0.001	<0.001	
**factor 1 x factor 2**	<0.001	<0.001		<0.001	<0.001	

Mean values ± standard deviation in column marked with the same superscript do not differ significantly at significance level of 0.05. Factor 1—type of maltodextrin in the emulsions system, Factor 2—dextrose equivalent DE, Factor 1 × factor 2—interactions between maltodextrin type and dextrose equivalent.
